# Genome-Wide DNA Methylation Profiling in CD8 T-Cells and Gamma Delta T-Cells of Asian Indian Patients With Takayasu Arteritis

**DOI:** 10.3389/fcell.2022.843413

**Published:** 2022-06-23

**Authors:** Jayakanthan Kabeerdoss, Debashish Danda, Ruchika Goel, Hindhumathi Mohan, Sumita Danda, R. Hal Scofield

**Affiliations:** ^1^ Department of Clinical Immunology and Rheumatology, Christian Medical College, Vellore, India; ^2^ Department of Pediatrics, Post Graduate Institute of Medical Education and Research (PGIMER), Chandigarh, India; ^3^ Department of Medical Genetics, Christian Medical College, Vellore, India; ^4^ Arthritis and Clinical Immunology Research Program, Oklahoma Medical Research Foundation, Oklahoma City, OK, United States; ^5^ Department of Veterans Affairs, University of Oklahoma Health Sciences Center, Oklahoma City, OK, United States

**Keywords:** DNA methylation, epigenetics, large vessel vasculitis, Takayasu’s arteritis, Interleukin-32

## Abstract

**Background:** Takayasu’s Arteritis (TA) is a chronic inflammatory disease that affects aorta and its main branches at their origin. Genetic, pathological and functional studies have shown that CD8 and Gamma delta (γ/δ) T-lymphocytes are involved in inflammatory processes in affected regions of arteries causing vascular damage. The molecular function of these lymphocytes remains unclear and currently no epigenetic studies are available in TA. We primarily performed genome wide methylation analysis in CD8 T cells and γδ T cells of patients with TA and compared with healthy controls.

**Methods:** We recruited 12 subjects in each group namely TA patient and healthy controls. Blood samples were collected after obtaining informed written consent. CD8 T cells and γδ T cells were separated from whole blood. DNA extracted from these cells and were subjected to bisulfite treatment. Finally, bisulfite treated DNA was loaded in Infinium Methylation EPIC array. Bioinformatics analysis was used to identify differential methylation regions which were then mapped to genes.

**Results:** Interleukin (IL)-32 and Lymphotoxin-A were genes significantly hypomethylated in CD8 T-cells. Anti-inflammatory cytokine genes, *IL-10*, *IL-1RN* and *IL-27* were hypomethylated in γδ T cells of TA patients as compared to healthy controls. Gene enrichment analysis using Gene Ontology (GO) database and Kyoto Encyclopaedia of Genes and Genomes (KEGG) identified that genes involved in T-cell receptor signalling pathways were hypomethylated in CD8 T-cells and hypermethylated in γδ T cells of TA patients.

**Conclusion:** CD8 T-cells might play a major role in immunopathogenesis of inflammation in TA, whereas γδ T cells may play a regulatory role.

• This maiden genome-wide DNA-methylation study in TA revealed hypomethylation of genes, *IL-32* and *LTA* in CD8+T-cells
• Anti-inflammatory cytokine genes *IL-10*, *IL-1RN* and *IL-27* were hypomethylated in γδ+ T-cells of TA patients
• Genes involved in T-cell receptor signalling pathways were hypomethylated in CD8 T-cells of TA patients

## Introduction

Takayasu’s Arteritis (TA) is an idiopathic chronic inflammatory disease that affects the aorta and its main branches at origin. TA is predominantly seen in women of reproductive age group and the onset is before the age of 40 in majority of cases. Aetiology of the TA is unknown. Pathophysiology of TA involves infiltration of leukocytes in vascular tissues involving all layers of large arteries. It is characterized by adventitial thickening, focal leukocyte infiltration of tunica media and intimal hyperplasia.

CD8 T-cells are in excess both in peripheral blood and inflamed vessels of patients with TA compared to giant cell arteritis (GCA) (Kurata et al., 2019; Matsumoto et al., 2019). In fact, even after treatment with biologic disease-modifying antirheumatic drugs (DMARDs), CD8 T-cells were not lowered in TA. This is in contrast to Th1, Th17, and Tfh cells, all of which are shown to be reduced in number after such therapy. High levels of CD8 T-cells are also reported to be associated with relapse in TA (Matsumoto et al., 2019). Both HLA-DR expressing CD8 and CD4^+^ T cells were increased in peripheral blood of patients with TA (Nityanand et al., 1997). Several other studies have shown that CD8^+^ T cells are involved in pathogenesis of TA by secreting specific cytokines and chemokines (Uppal and Verma, 2003; Régnier et al., 2020).

γ/δ T cells represent 1-5% of peripheral blood T cells. Seko et al. found that γ/δ T cells contribute around 30% of leukocytes infiltrating aortic tissues of TA (Seko et al., 1996). Aortic tissues responding to unknown stimulus express 65 kDa heat-shock protein, which in turn induce expression of MHC- I–related chain A (MICA) on the surface of vascular smooth muscle cell (VSMC). MICA on VSMC is recognized by NKG2D receptors in γ/δ T cells and CD8^+^ T cells, which secrete cell granules containing perforin and interferon resulting in initiation of vascular inflammation (Arnaud et al., 2011). Though the above mentioned published data demonstrated involvement of CD8 T cells and γ/δ T cells in TA, their pathogenic roles are not yet fully understood. The current study aimed to explore genome wide DNA methylation changes in CD8 T cells and γδ T cells of patients with TA in comparison with healthy individuals as controls.

## Methods

### Patients and Controls

Twelve patients satisfying ACR 1990 criteria for TA were recruited from Rheumatology clinics of Christian Medical College, Vellore (Arend et al., 1990). Age and sex matched healthy subjects also recruited as controls for the study. Participants were recruited between September 2015 and January 2016 after obtaining written informed consent. The study followed the tenets of the Declaration of Helsinki and was approved by the Institutional review board of Christian Medical College, Vellore.

### Cell Separation, DNA Extraction, and Bisulfite Conversion

Twenty ml of whole blood were collected from each participant in anticoagulated vacutainer tubes. Peripheral blood mononuclear cells (PBMCs) were isolated from whole blood by density gradient centrifugation using Ficoll-PaqueTM Plus (Catalogue no. 1033378, GE Healthcare). CD8 and γδ T cells were separated from PBMC using magnetic labelling based separation methods. CD8 microbeads (Catalogue no. 130-045-201, Miltenyi Biotec, CA, USA) was used for separation of CD8 T cells by negative selection method, followed by use of Anti-TCR γδ microbead kit (catalog no. 130-050-701, Miltenyi Biotec, CA, USA) for separation of γδ T cells. DNA was extracted from these cells using the DNeasy Blood and Tissue Kit (Qiagen, Valencia, CA) according to the manufacturer’s protocols. DNA samples were stored at −80°C until processing of methylation profiling was done.

### DNA Methylation Profiling

Genomic DNA samples of both CD8 and γδ T cells from each participant were subjected to bisulfite treatment using the EZ DNA Methylation-Gold Kit (Zymo Research, Orange, CA). Infinium Methylation EPIC arrays (Illumina, San Diego, CA) were used to assess the genome-wide DNA methylation levels. This chip array allows for the interrogation of over 850,000 methylation sites within the entire genome covering CpG islands, genes, and enhancers, DNase hypersensitive sites and miRNA promoter regions. All array handling, sample hybridization, and array scanning were performed at a commercial service provider lab (M/s. Medgenome labs Pvt Ltd., Bengaluru, India). The service provider was blinded to the source of the samples. Raw idat and sample annotation files were received from a service provider for bioinformatics analysis.

### Bioinformatics Analysis for Methylation Data

Data from methylation chip array were analysed in R software ChAMP package (Morris et al., 2014). Raw idat files and sample annotation were uploaded in ChAMP using champ.import function. Default filter function was used to remove low quality probes. This includes removal of each probe having *p*-value above 0.01, non-CpG probes, all SNP-related probes and all probes located on chromosome X or Y. Normalisation of data was done for adjustment of bias in the type-II probe (Supplementary Figure S1 for before and after normalisation of probes). Samples were run in chip array on different batches. For removal of the batch effect, correction was performed in ChAMP package. Differential methylation probes (DMP) and Differential methylation region (DMR) analysis were performed between TA and healthy controls. DMR analysis were performed separately in the DMRcate package from the Bioconductor platform in R, as this function within the ChAMP pipeline was not functional (Peters et al., 2015). Beta (*β*) value is the ratio of methylated intensity and the overall intensity values. Normalised Beta values of each probe were extracted from ChAMP pipeline and loaded in DMRcate package to identify quantitative alteration in DNA methylation levels between cases and controls.

Gene enrichment analysis were performed for genes significant in DMR using cluster Profiler package in R with *p*-value cutoff of <0.05(Yu et al., 2012). Significant genes from DMR were used after converting gene symbols to entrez gene id in the online tool DAVID (https://david.ncifcrf.gov). Gene Ontology (GO) enrichment analysis was performed to identify over-represented GO terms with combined domains of biological processes, molecular function and cellular components. KEGG (Kyoto Encyclopaedia of Genes and Genomes) pathway gene set enrichment (GSE) analysis was performed by employing a hypergeometric test within cluster Profiler package. Significant gene enrichment were visualised by network and pathway based plots using enrichplot and pathview packages respectively in R Bioconductor tool.

## Results

### TA Patient and Control Characteristics

Demographic and clinical details of TA patients are provided in Table 1. Median age of healthy controls was 23 (15-48) years and Female: male ratio was 10:2. Both age and sex ratio were matched for cases and controls.

**TABLE 1:** Clinical details of patients with TA.

Parameter	*n* = 12
Gender (Male: Female)	2:10
Median age in years (range)	26 (18–39)
Median duration of symptoms in months (range)	18 (0–48)
Angiographic types n(%)	
Type I	1 (8.3%)
Type IIb	1 (8.3%)
Type III	1 (8.3%)
Type IV	1 (8.3%)
Type V	8 (66.6%)
Median ESR in mm/1st hour (range)	42.8 (6–75)
Median CRP mg/dl (range)	31.6 (3–87)
Median ITAS 2010 (range)	8.6 (0–17)
Median ITAS -CRP(range)	10.4 (2–20)
Median DEI.Tak (range)	9.5 (4–13)
Treatment details	N (%)
Treatment naïve	8 (66.7%)
Glucocorticoids	2 (16.7%)
Defaulters of treatment	2 (16.7%)
Biological DMARDs	Nil

### Differential Methylated CpG Sites in TA

**FIGURE 1:**
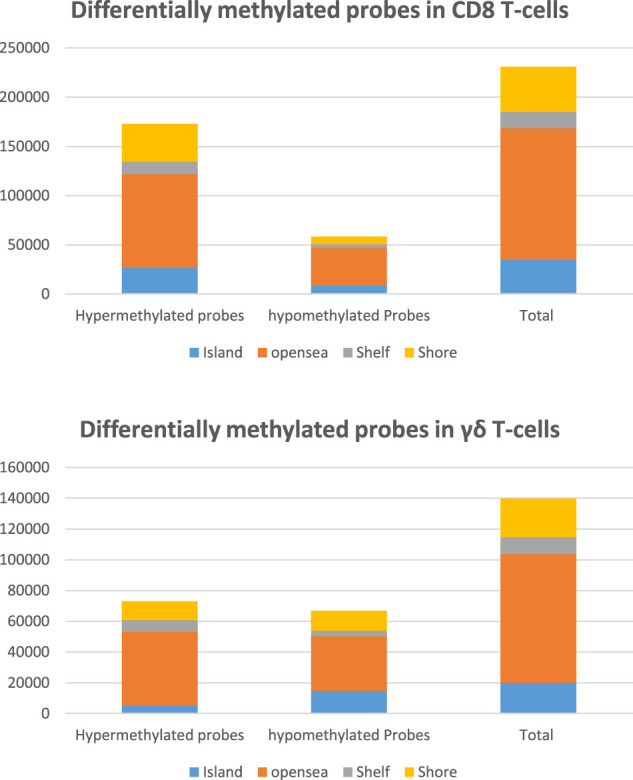
Differentially methylated CpG sites in CD8 T cells and γδ T cells with p-values < 0.05.

Total 850 K probes were available in the chip array. After quality control and filtering of probes nearly 700 K probes were available for analysis for both CD8 T cells and γδ T cells to examine differential methylation CpG probes between TA and healthy control. Number of significantly hypermethylated and hypomethylated CpG sites for CD8 T cells and γδ T cells are depicted in Figure 1.

### Differential Methylated Regions in TA

Nearly 34000 regions were differentially methylated in CD8 T cells between TA and controls which comprised 221665 CpG sites (probes) with a threshold FDR <0.05, including 27243 hypermethylated regions and 7104 hypomethylated regions. In γδ T cells, 24088 regions were differentially methylated comprising of 115427 CpG sites, which included 10834 hypermethylated regions and 13255 hypomethylated regions. Top 20 genes containing hypermethylated and hypomethylated regions for CD8 T cells and γδ T cells were listed in Supplementary Tables S1, S2 respectively.

**FIGURE 2:**
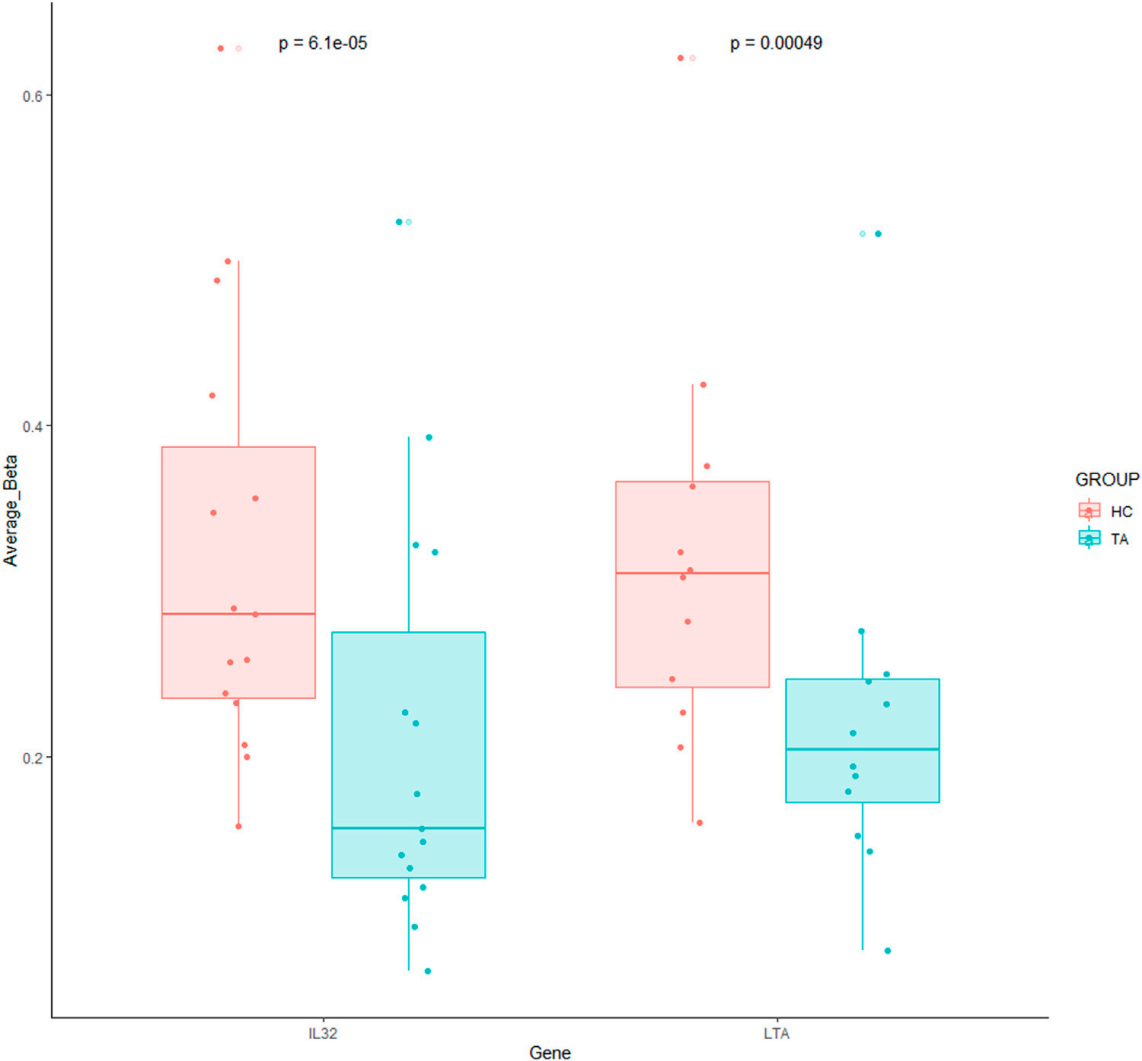
Significantly hypomethylated genes in CD8 T-cells of patients with TA. Each dot indicates each CpG probe measured within this gene. A Wilcoxon matched paired test was used to compare between the groups against each CpG probe.

**FIGURE 3:**
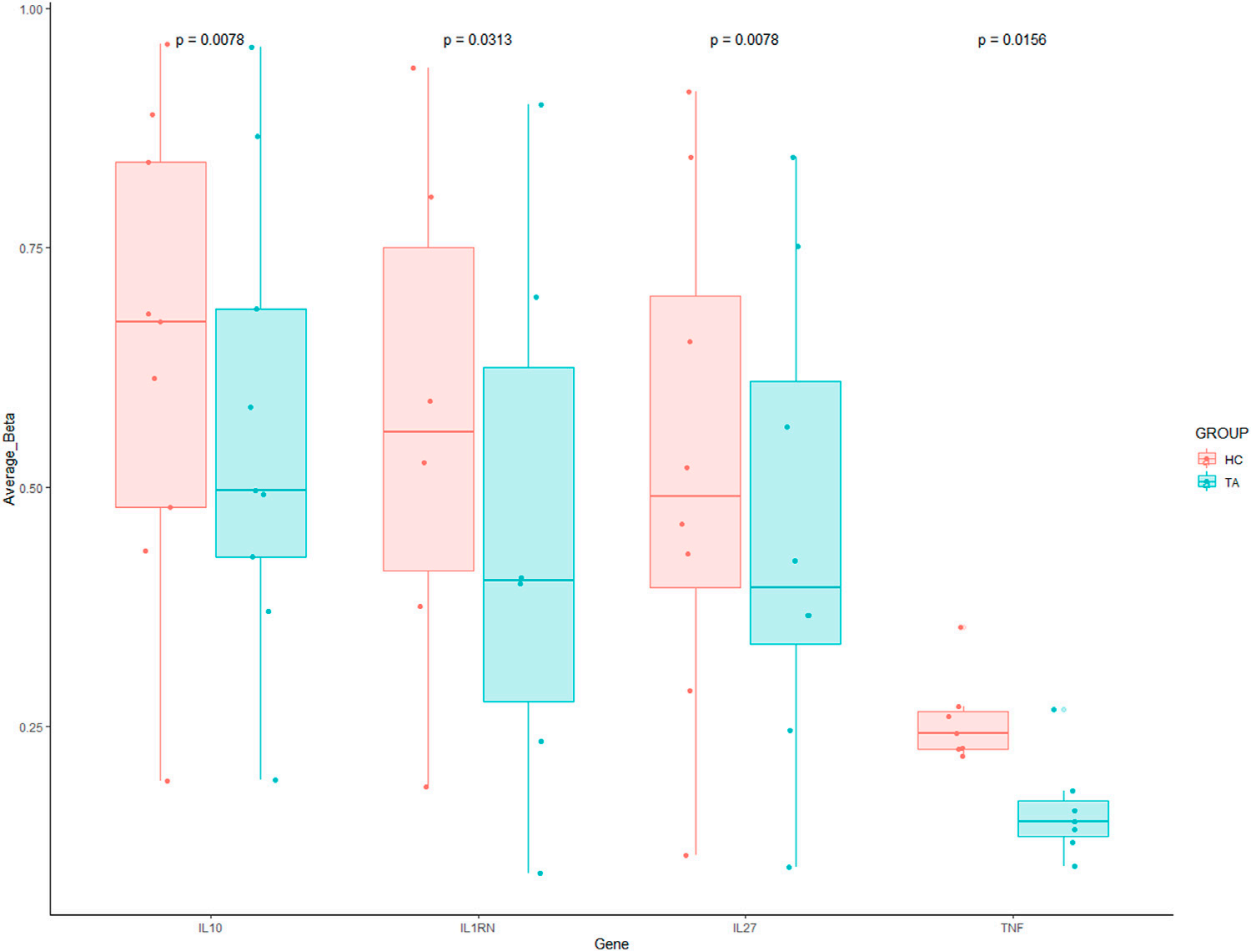
Significantly hypomethylated genes in γδ T cells of patients with TA. Each dot indicates each CpG probe measured within this gene. A Wilcoxon matched paired test was used to compare between the groups against each CpG probe.

Hypomethylated regions are associated with increased gene expression, especially in pro-inflammatory cytokine genes, which are of interest in inflammatory diseases. In CD8 T-cells, Interleukin-32 (*IL-32*) and Lymphotoxin -Alpha (*LTA*) were significantly hypomethylated genes in our TA patients compared to healthy controls (Figure 2). *TNF-α*, *IL-10* and *IL-27* genes were significantly hypomethylated in γδ T cells of our TA patients (Figure 3).

**FIGURE 4:**
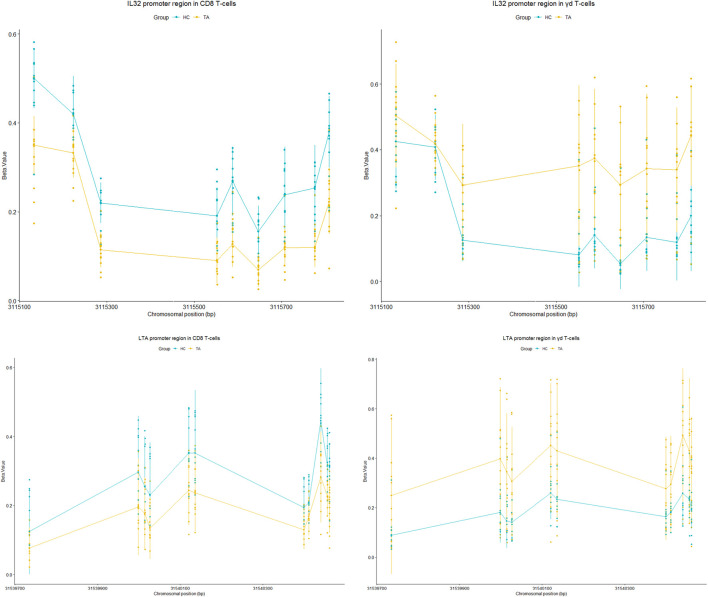
Tukey plot showing significantly differentially methylated regions (CpG sites) in promoter region of *IL-32* (chr 16: 3114847–3115809) and *LTA* (chr 6: 31539539- 31541349) genes in CD8 T cells and γδ T cells of TA as well as healthy controls. Both genes were hypomethylated in CD8 T cells and hypermethylated in γδ T cells of TA as compared to healthy controls.

**FIGURE 5:**
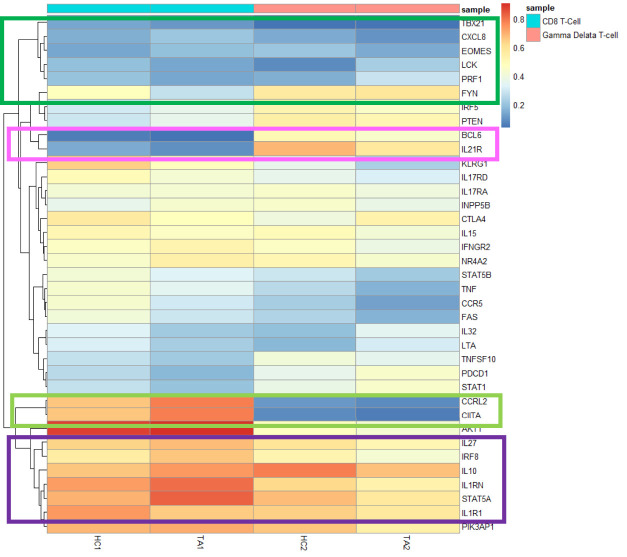
Heatmap plot showing hypomethylated genes in CD8 T cells and γδ T cells of TA patients as compared with healthy controls. *BCL6* and *IL21R* were hypomethylated genes in only CD8 T-cells (marked in pink colour box). *CCRL2* and *CIITA* genes were hypomethylated in only γδ T-cells (marked in light green colour box).

We, therefore, observed differential methylation of regions between CD8 and γδ T cells in patients with TA. *LTA* and *IL-32* were significantly hypomethylated in CD8 T-cells, whereas these genes were hypermethylated in γδ T cells on TA patients (Figure 4). This shows cell specific epigenetic changes in patients with TA. Cell specific methylation pattern is better visualised in heatmap plot (Figure 5). *BCL6* and *IL21R* are also hypomethylated in CD8 T-cells but hypermethylated in γδ T cells. Inversely, *CCRL2* and *CIITA* genes are hypomethylated in γδ T-cells while hypermethylated in CD8 T-cells in TA patients.

Our study also revealed hypomethylation of genes involved in transcription factors namely, *TBX21* and *EOMES* in CD8 T cells and γδ T cells (Figure 5), However, significant hypomethylation for *TBX21* was found only in CD8 T cells and the same for *EOMES* was documented only in γδ T cells of our patients with TA. Genes of proteins involved in T-cell receptor (TCR) signalling and perforin-1 were hypomethylated in CD8 T cells at higher levels than γδ T cells. In contrast, genes of anti-inflammatory cytokines, *IL-10*, *IL-1RN*, *IL27* and transcription factor *STAT5A* were hypomethylated at lower levels in CD8 T cells compared to γδ T cells of patients with TA (Figure 5).

### DMR Associated Functional Pathways in TA

Having established sets of genes from DMR, we identified function pathways using GO resources and KEGG database. A list of significant pathways in the GO and KEGG database for CD8 T cells and γδ T cells were mentioned in Supplementary Tables S3, S4 respectively.

Gene ontology (GO) analyses in CD8 T cells revealed neutrophil mediated immunity, neutrophil activation, neutrophil degranulation, and lymphocyte degranulation pathways were hypermethylated in TA, whereas ribosome structure, viral transcription and viral gene expression were hypomethylated (Supplementary Figure S2). These findings were confirmed in KEGG enrichment analysis that genes involved in ribosome and T-cell receptor signalling were hypomethylated (Supplementary Figure S2) in CD8 T cells of our TA patients.

GO analyses in γδ T cells of TA showed opposite patterns compared to CD8 T-cells. Myeloid cell activation, neutrophil activation, neutrophil degranulation, lymphocyte degranulation were hypomethylated and TCR signalling pathway, antigen-receptor mediated signalling, T-cell activation, T-cell differentiation and adaptive immune response pathways were hypermethylated (Supplementary Figure S3). Again, this findings was confirmed in KEGG GSE analysis that TCR signalling pathway, Th17 differentiation and antigen processing and presentation were hypermethylated in γδ T cells of patients with TA (Supplementary Figure S3).

**FIGURE 6:**
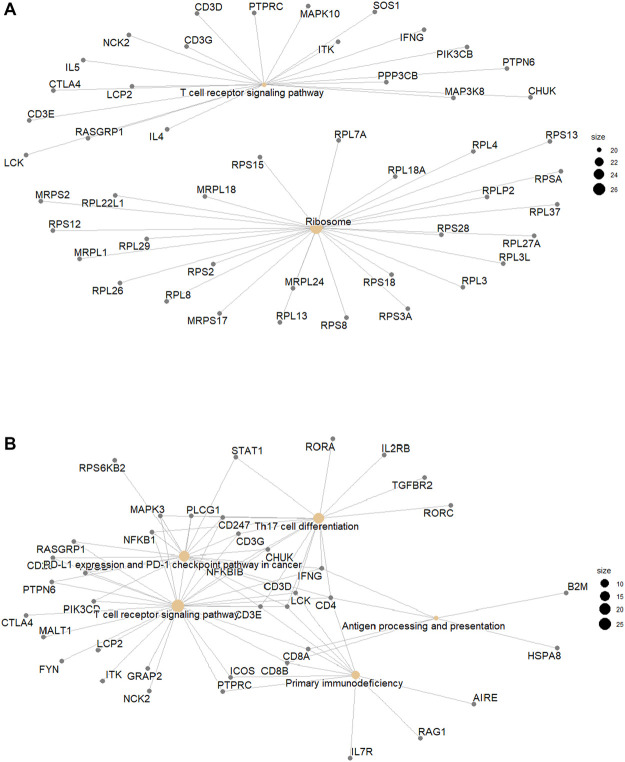
**(A)**. Hypomethylated genes in CD8 T cells and **(B)**. Hypermethylated Genes in γδ T cells by KEGG pathways.

Genes involved in statistically significant pathways identified by KEGG analysis for both CD8 T cells and γδ T cells were visualised in network plot (Figure 6). Hypomethylated and hypermethylated genes involved in TCR signalling were visualised in pathway plot (Supplementary Figure S4).

## Discussion

To our knowledge, this is the first study describing the methylation changes in CD8 T cells and γδ T cells of patients with TA in comparison with healthy controls. Our study showed *IL-32* and *LTA* genes were significantly hypomethylated in CD8 T-cells from patients with TA. Also *TNF-α*, *IL-10* and *IL-27* genes were significantly hypomethylated in γδ T cells of TA patients. Another important finding is that genes involved in T-cell receptor signalling were hypomethylated in CD8-T cells from patients with TA.

In our study, the promoter region of *IL-32* gene is significantly hypomethylated in patients with TA compared to healthy controls. Increased *IL-32* expression and serum levels has been reported in patients of GCA and Anti-Neutrophilic Cytoplasmic Autoantibody (ANCA) associated vasculitis(Ciccia et al., 2011; Bae et al., 2012; Krajewska Wojciechowska et al., 2019). In GCA, *IL-32* expression is found in inflammed vessels and it co-localises with Th1 lymphocytes (Ciccia et al., 2011). *IL-32* expressed by CD8 T-cells was reported to be associated with history of Polymyalgia Rheumatica (PMR) and abnormal neutrophil count in patients with GCA (De Smit et al., 2018). *IL-32* induces dendritic cells to secrete the chemokine *RANTES* (also known as CCL5), which in turn recruits activated T-cells expressing *CCR5* (Figure 5) to inflammatory sites and thereby causes vascular dysfunction (Son et al., 2014; Mikolajczyk et al., 2016). Again, in our study *CCR5* is also hypomethylated in CD8 T-cells of TA. This shows IL-32 might contribute to activation and recruitment of CD8 T-cells in TA.

In the present study, another important cytokine gene LTA encoding lymphotoxin-A, previously known as TNF-beta is significantly hypomethylated in CD8 T cells of patients with TA. An earlier study in GCA showed lymphotoxin expression localised with formation of tertiary lymphoid organs (TLOs) in inflamed arteries (Ciccia et al., 2017). *LTA* gene is shown to be hypomethylated in CD4 T-lymphocytes of patients with primary Sjögren’s syndrome (pSS) (Altorok et al., 2014). Again, TLOs were the source of autoreactive lymphocytes in inflamed regions of salivary glands of pSS (Asam et al., 2021). Can this suggest that LTA secreted by CD8 T-cells may be involved in formation of TLOs in inflamed arteries of patients with TA?

In Bechet’s disease, another vasculitis involving large vessels in vast majority of them, γδ T cells are shown to secrete TNF-α and CXCL8 causing activation signal and recruitment of neutrophils and monocytes to sites of infection and inflammation (Hasan et al., 2015). This is similar to the findings in our study showing hypomethylation of *TNF-α* and *CXCL8* genes in γδ T cells of TA. In addition, anti-inflammatory cytokines *IL-10*, *IL-1RN* and *IL-27* genes were also hypomethylated. This is again similar to previous findings in patients with BD that reported γδ T cells predominantly as regulatory in nature and secrete lower levels of inflammatory cytokines (Parlakgul et al., 2013).

Seko et al. showed that aortic tissues express 65-kD heat-shock protein (HSP-65), which was recognised by infiltrating killer lymphocytes resulting in secretion of perforin, which led to vascular cell injury of Takayasu’s arteritis (Seko et al., 1994). Chauhan et al. demonstrated this cytotoxic function of lymphocytes to be mediated by interaction of Fas and FasL as well as with the help of secretion of IFN-γ (Chauhan et al., 2006; Chauhan et al., 2007). Our findings concur with the findings of that study as perforin-1(*PRF1*), *Fas* and *IFN-γ* genes were significantly hypomethylated in CD8 T-cells, but not in γδ T cells of TA (Figure 5). In addition, we didn’t find any hypomethylation of *HSP65* gene; rather it was noted in genes of *HSPA1A*, *HSPA1L*, both expressing 70-kd heat-shock protein (data not shown). Thus CD8 T-cells might be more cytotoxic in nature and involved in vascular cell injury in patients of TA.

On the other hand, Transcription factor T-bet encoded by gene *TBX21* is required for differentiation of effector CD8 T-cells producing INF-γ following encounter with self-antigens (Jackson et al., 2014). But, Eomes expression in γδ T cells leads to differentiation of Th1-like lymphocytes producing IFN-γ (Lino et al., 2017). Thus hypomethylation of *TBX21* and *EOMES* in CD8 T-cells and γδ T cells might be attributed to IFN-γ secreting Th1 like subsets in patients with TA; however, it is difficult to conclude this point at this stage and further confirmation by future studies may be needed. *CTLA4* and *IL-21R* were also hypomethylated genes in patients with TA, which are markers of regulatory and follicular T-cells subsets. This shows that different subsets exist amongst CD8 lymphocytes in TA.

Gene enrichment analysis in CD8 T-cells of TA patients showed hypomethylation of genes involved in T-cell receptor signalling. McKinney et al. demonstrated that TCR signalling is most pronounced in effector-memory (T_EM_) subset of CD8 T-cells in patients with ANCA vasculitis (McKinney et al., 2010). *LCK* and *PRF-1* genes were significantly hypomethylated in CD8 T-cells, whereas these genes were hypermethylated in γδ T cells of TA in this study. LCK is a tyrosine kinase essential for downstream signalling of activated T-lymphocytes. In CD8 T-cells, absence of LCK results in reduced perforin mediate killing, thereby affect its cytotoxic function (Milstein et al., 2011). This shows that activated CD8 T-cells of patients with TA have higher cytotoxic ability as compared to healthy controls.

In the current study, genes involved in TCR signalling pathway and Th17 differentiation were hypermethylated in γδ T cells of TA. Evidence from literature suggest that γδ T cells can produce IL-17 in response to IL-1β and IL-23. Activation of TCR in γδ T cells leads to differentiation of IL-17 producing cells (Akitsu and Iwakura, 2018). This suggests that γδ T cells might not be the source of IL-17 in patients with TA as reported earlier (Misra et al., 2016).

This study is not without limitations. We didn’t check purity of CD8 T cells and γδ T cells after separation of these cells from PBMC. However, as per the brochure of the magnetic separation kit used in our study, it is expected to achieve greater than 97% purity. Another important limitation of our study: we didn’t perform validation assays such as combined bisulfite restriction analyses or pyrosequencing to confirm the findings of the present study.

Strength of our study is the novelty of being the first ever report on genome wide methylation profiling in CD8 T cells and γδ T cells in patients with TA. Measurement of the expression levels of IL-32 and LTA in CD8 T-cells as well as TNF-α, IL-10 and IL-27 in γδ T cells from patients with TA using flow cytometry analysis may be used in future studies, to explore if these cytokines can be used as diagnostic or prognostic biomarkers.

## Conclusion

Our study showed that *IL-32* and *LTA* were significantly hypomethylated in CD8 T-cells and anti-inflammatory cytokine genes *IL-10*, *IL-27* and *IL-1RN* were significantly hypomethylated in γδ T cells of TA. Genes involved in TCR signalling pathway and ribosome were also significantly hypomethylated in CD8 T-cells. Genes involved in TCR signalling pathway and Th17 differentiation, on the contrary, were hypermethylated in γδ T cells from patients with TA. Overall evidence from this study, and in the light of the published literature, emphasises that CD8 T-cells are likely to be more crucially involved in pathogenesis of TA, rather than γδ T cells.

## Data Availability

The raw data supporting the conclusions of this article will be made available by the corresponding author on request.
