# Supplementary material for: Genome-Wide DNA Methylation Profiling in CD8 T-Cells and Gamma Delta T-Cells of Asian Indian Patients With Takayasu Arteritis
Source: Front Cell Dev Biol. 2022 Jun 23;10:843413. doi: 10.3389/fcell.2022.843413 (PMC9259853; doi:10.3389/fcell.2022.843413)
Supplement: Supplementary file 1 [file Table1.DOCX]

**Supplementary Table -1:** Top 20 genes differentially methylated regions between TA and Healthy controls in CD8 T-cells

| **Chromosome** | **start** | **end** | **width strand** | **no.cpgs** | **min_smoothed_fdr** | **Stouffer** | **HMFDR** | **Fisher** | **maxdiff** | **meandiff** | **overlapping.genes** |
| --- | --- | --- | --- | --- | --- | --- | --- | --- | --- | --- | --- |
| chr8 | 38830814 | 38832728 | 1915 | 15 | 1.75E-97 | 1.21E-40 | 4.65E-05 | 5.90E-37 | 0.199555 | 0.122175604 | PLEKHA2 |
| chr14 | 1.06E+08 | 1.06E+08 | 2920 | 19 | 1.69E-89 | 2.18E-51 | 4.73E-05 | 5.87E-47 | 0.18077 | 0.134673516 | IGHJ6 |
| chr6 | 32114325 | 32123701 | 9377 | 117 | 7.09E-70 | 3.23E-52 | 0.000755 | 2.08E-58 | -0.18722 | 0.02886992 | PPT2 |
| chr4 | 1.55E+08 | 1.55E+08 | 5097 | 42 | 6.64E-67 | 9.83E-34 | 0.000898 | 1.23E-35 | 0.150521 | 0.05229066 | SFRP2 |
| chr17 | 37893638 | 37897076 | 3439 | 26 | 1.45E-62 | 1.11E-38 | 0.000363 | 1.10E-38 | 0.143845 | 0.078448021 | GRB7 |
| chr18 | 49865488 | 49869484 | 3997 | 33 | 1.10E-53 | 2.80E-35 | 0.00109 | 2.19E-33 | 0.124101 | 0.051690967 | DCC |
| chr5 | 1.19E+08 | 1.19E+08 | 2981 | 24 | 6.61E-50 | 2.04E-27 | 0.000258 | 8.30E-26 | -0.22647 | -0.087780666 | TNFAIP8 |
| chr16 | 3114847 | 3115809 | 963 | 12 | 3.58E-48 | 8.13E-23 | 0.000602 | 1.35E-21 | -0.1597 | -0.101671966 | IL32 |
| chr11 | 27739827 | 27745361 | 5535 | 30 | 4.99E-41 | 1.76E-35 | 0.000259 | 4.55E-32 | 0.116047 | 0.04465174 | BDNF |
| chr2 | 1.57E+08 | 1.57E+08 | 4635 | 22 | 8.22E-41 | 2.27E-43 | 0.000302 | 2.93E-37 | 0.164168 | 0.10896639 | NR4A2 |
| chr11 | 1.18E+08 | 1.18E+08 | 1841 | 10 | 5.39E-37 | 2.01E-26 | 0.000293 | 3.82E-23 | -0.1374 | -0.11280219 | CD3G |
| chr6 | 33171467 | 33181870 | 10404 | 106 | 1.26E-36 | 2.77E-40 | 0.002516 | 2.38E-40 | -0.13403 | 0.000111071 | SLC39A7 |
| chr11 | 1.29E+08 | 1.29E+08 | 9173 | 36 | 3.17E-32 | 7.11E-36 | 0.001019 | 1.41E-33 | 0.175148 | 0.061205585 | FLI1 |
| chr5 | 50673033 | 50679965 | 6933 | 40 | 7.52E-32 | 2.21E-34 | 0.000653 | 5.83E-32 | 0.134125 | 0.044287485 | ISL1 |
| chr17 | 38023480 | 38024636 | 1157 | 7 | 1.42E-27 | 9.38E-15 | 0.000414 | 3.35E-13 | -0.14997 | -0.092054723 | ZPBP2 |
| chr5 | 1.32E+08 | 1.32E+08 | 1478 | 9 | 6.87E-27 | 3.28E-15 | 0.000817 | 7.89E-14 | -0.23773 | -0.101906394 | IL5 |
| chr2 | 2.31E+08 | 2.31E+08 | 2481 | 11 | 3.18E-26 | 7.52E-14 | 0.001698 | 8.01E-14 | -0.13521 | -0.084867673 | SP140 |
| chr6 | 31539539 | 31541349 | 1811 | 18 | 9.04E-26 | 5.97E-15 | 0.009922 | 3.27E-13 | -0.14876 | -0.077467634 | LTA |
| chr2 | 1.01E+08 | 1.01E+08 | 1677 | 7 | 5.09E-24 | 3.28E-13 | 9.88E-05 | 3.60E-13 | -0.30859 | -0.072329785 | NMS |

**Supplementary Table -2.** Top 20 genes differentially methylated regions between TA and Healthy controls in γδ T-cells

| **seqnames** | **start** | **end** | **width** | **no.cpgs** | **min_smoothed_fdr** | **Stouffer** | **HMFDR** | **Fisher** | **maxdiff** | **meandiff** | **overlapping.genes** |
| --- | --- | --- | --- | --- | --- | --- | --- | --- | --- | --- | --- |
| chr6 | 31650735 | 31651676 | 942 | 19 | 1.40E-27 | 0.078734 | 0.319267 | 0.331969 | -0.27088534 | -0.13009 | LY6G5C |
| chr20 | 57581903 | 57583709 | 1807 | 27 | 2.05E-17 | 0.231775 | 0.371012 | 0.645531 | -0.210828856 | -0.09162 | CTSZ |
| chr6 | 30042260 | 30045280 | 3021 | 37 | 6.63E-15 | 0.737203 | 0.435181 | 0.953836 | 0.095418705 | -0.01884 | RNF39 |
| chr10 | 51571362 | 51572718 | 1357 | 13 | 1.14E-12 | 0.181421 | 0.334162 | 0.414828 | -0.100577609 | -0.05986 | NCOA4 |
| chr3 | 48699637 | 48701770 | 2134 | 17 | 4.18E-12 | 0.290316 | 0.375506 | 0.595177 | -0.051941509 | -0.01865 | RP11-148G20.1, CELSR3, NCKIPSD |
| chr19 | 17515486 | 17517762 | 2277 | 13 | 5.30E-12 | 0.042911 | 0.309724 | 0.262492 | -0.105244128 | -0.06047 | CTD-2521M24.9, MVB12A, BST2 |
| chr6 | 28910953 | 28912166 | 1214 | 14 | 3.92E-10 | 0.371726 | 0.366361 | 0.564796 | -0.080404264 | -0.03571 | C6orf100 |
| chr2 | 1.28E+08 | 1.28E+08 | 606 | 10 | 4.86E-10 | 0.058302 | 0.304929 | 0.262178 | -0.170352595 | -0.11525 | SFT2D3, WDR33 |
| chr8 | 1.26E+08 | 1.26E+08 | 1987 | 14 | 5.64E-10 | 0.976849 | 0.514977 | 0.980762 | -0.094232487 | -0.01928 | SQLE |
| chr1 | 1.67E+08 | 1.67E+08 | 1999 | 9 | 1.22E-07 | 0.043507 | 0.28403 | 0.204409 | 0.207924098 | 0.15344 | CD247 |
| chr11 | 1.18E+08 | 1.18E+08 | 1841 | 10 | 1.49E-07 | 0.032465 | 0.279711 | 0.183696 | 0.227063971 | 0.183629 | CD3G, CD3D |
| chr12 | 56329039 | 56329903 | 865 | 6 | 2.22E-07 | 0.075277 | 0.278639 | 0.223675 | 0.227195229 | 0.178794 | DGKA |
| chr17 | 1774149 | 1775372 | 1224 | 6 | 3.01E-06 | 0.077174 | 0.280426 | 0.227709 | 0.222968502 | 0.194796 | RPA1 |
| chr15 | 45006400 | 45007854 | 1455 | 5 | 3.40E-06 | 0.094706 | 0.278639 | 0.236329 | 0.212975793 | 0.140044 | B2M |
| chr14 | 23015364 | 23016326 | 963 | 6 | 9.08E-06 | 0.078777 | 0.281888 | 0.231071 | 0.31086155 | 0.279415 | NA |
| chr18 | 3250274 | 3252175 | 1902 | 6 | 9.79E-06 | 0.083257 | 0.285395 | 0.239928 | 0.179234668 | 0.137584 | MYL12A |
| chr1 | 24832616 | 24834209 | 1594 | 6 | 2.65E-05 | 0.081364 | 0.284116 | 0.236368 | 0.189171779 | 0.150386 | RCAN3 |
| chr21 | 47844012 | 47846235 | 2224 | 8 | 4.62E-05 | 0.058015 | 0.288005 | 0.225926 | 0.214235119 | 0.166729 | PCNT |
| chr7 | 99220820 | 99222459 | 1640 | 5 | 0.000207 | 0.096585 | 0.280274 | 0.239799 | 0.200280212 | 0.157539 | ZSCAN25 |

**Supplementary Table -3a**: Significant pathways between TA and Healthy controls in CD8 T-cells identified by GO database

| **ONTOLOGY** | **ID** | **Description** | **setSize** | **enrichmentScore** | **NES** | **pvalue** | **p.adjust** | **qvalues** |
| --- | --- | --- | --- | --- | --- | --- | --- | --- |
| CC | GO:0022626 | cytosolic ribosome | 63 | -0.54577 | -2.39475 | 2.35E-08 | 0.000165 | 0.000153 |
| BP | GO:0006614 | SRP-dependent cotranslational protein targeting to membrane | 55 | -0.55026 | -2.41896 | 1.01E-07 | 0.000355 | 0.00033 |
| BP | GO:0019083 | viral transcription | 110 | -0.39992 | -1.96378 | 8.27E-07 | 0.001935 | 0.001798 |
| CC | GO:0044391 | ribosomal subunit | 107 | -0.39723 | -1.92727 | 1.64E-06 | 0.002883 | 0.002679 |
| BP | GO:0019080 | viral gene expression | 121 | -0.3692 | -1.83353 | 2.25E-06 | 0.00312 | 0.002899 |
| BP | GO:0006413 | translational initiation | 108 | -0.38467 | -1.84327 | 2.70E-06 | 0.00312 | 0.002899 |
| BP | GO:0036230 | granulocyte activation | 313 | 0.426647 | 1.485422 | 3.11E-06 | 0.00312 | 0.002899 |
| BP | GO:0042119 | neutrophil activation | 310 | 0.426362 | 1.484186 | 4.67E-06 | 0.004092 | 0.003803 |
| CC | GO:0022627 | cytosolic small ribosomal subunit | 26 | -0.65258 | -2.45023 | 5.75E-06 | 0.00448 | 0.004163 |
| MF | GO:0003735 | structural constituent of ribosome | 96 | -0.39288 | -1.9039 | 6.46E-06 | 0.00453 | 0.004209 |
| BP | GO:0002446 | neutrophil mediated immunity | 314 | 0.424336 | 1.477463 | 1.01E-05 | 0.006425 | 0.005971 |
| BP | GO:0002444 | myeloid leukocyte mediated immunity | 347 | 0.415245 | 1.44815 | 1.18E-05 | 0.006871 | 0.006385 |
| CC | GO:0015935 | small ribosomal subunit | 43 | -0.5354 | -2.15994 | 1.33E-05 | 0.006993 | 0.006499 |
| BP | GO:0006613 | cotranslational protein targeting to membrane | 58 | -0.47358 | -2.08565 | 1.40E-05 | 0.006993 | 0.006499 |
| BP | GO:0072599 | establishment of protein localization to endoplasmic reticulum | 68 | -0.42795 | -1.93077 | 1.95E-05 | 0.008619 | 0.00801 |
| BP | GO:0002283 | neutrophil activation involved in immune response | 305 | 0.421732 | 1.466702 | 2.03E-05 | 0.008619 | 0.00801 |
| BP | GO:0043299 | leukocyte degranulation | 333 | 0.412584 | 1.436152 | 2.20E-05 | 0.008619 | 0.00801 |
| BP | GO:0043312 | neutrophil degranulation | 303 | 0.423968 | 1.474726 | 2.22E-05 | 0.008619 | 0.00801 |
| BP | GO:0009617 | response to bacterium | 329 | 0.412681 | 1.436204 | 2.33E-05 | 0.008619 | 0.00801 |

**Supplementary Table -3b**: Significant pathways between TA and Healthy controls in CD8 T-cells identified by the KEGG database

| **ID** | **Description** | **GeneRatio** | **BgRatio** | **pvalue** | **p.adjust** | **qvalue** |
| --- | --- | --- | --- | --- | --- | --- |
| hsa04015 | Rap1 signaling pathway | 169/4708 | 210/8105 | 2.75E-12 | 7.84E-10 | 4.26E-10 |
| hsa04010 | MAPK signaling pathway | 226/4708 | 294/8105 | 4.77E-12 | 7.84E-10 | 4.26E-10 |
| hsa01521 | EGFR tyrosine kinase inhibitor resistance | 70/4708 | 79/8105 | 2.62E-09 | 2.10E-07 | 1.14E-07 |
| hsa04510 | Focal adhesion | 156/4708 | 201/8105 | 3.10E-09 | 2.10E-07 | 1.14E-07 |
| hsa04360 | Axon guidance | 143/4708 | 182/8105 | 3.19E-09 | 2.10E-07 | 1.14E-07 |
| hsa05224 | Breast cancer | 118/4708 | 147/8105 | 7.68E-09 | 3.94E-07 | 2.14E-07 |
| hsa04725 | Cholinergic synapse | 94/4708 | 113/8105 | 8.38E-09 | 3.94E-07 | 2.14E-07 |
| hsa04934 | Cushing syndrome | 122/4708 | 155/8105 | 3.84E-08 | 1.58E-06 | 8.59E-07 |
| hsa01522 | Endocrine resistance | 82/4708 | 98/8105 | 4.47E-08 | 1.63E-06 | 8.89E-07 |
| hsa05017 | Spinocerebellar ataxia | 113/4708 | 143/8105 | 8.25E-08 | 2.55E-06 | 1.39E-06 |
| hsa04152 | AMPK signaling pathway | 97/4708 | 120/8105 | 8.76E-08 | 2.55E-06 | 1.39E-06 |
| hsa05226 | Gastric cancer | 117/4708 | 149/8105 | 9.32E-08 | 2.55E-06 | 1.39E-06 |
| hsa04012 | ErbB signaling pathway | 72/4708 | 85/8105 | 1.09E-07 | 2.75E-06 | 1.49E-06 |
| hsa04929 | GnRH secretion | 56/4708 | 64/8105 | 2.92E-07 | 6.80E-06 | 3.70E-06 |
| hsa04072 | Phospholipase D signaling pathway | 115/4708 | 148/8105 | 3.36E-07 | 6.80E-06 | 3.70E-06 |
| hsa04921 | Oxytocin signaling pathway | 119/4708 | 154/8105 | 3.61E-07 | 6.80E-06 | 3.70E-06 |
| hsa04935 | Growth hormone synthesis, secretion and action | 95/4708 | 119/8105 | 3.70E-07 | 6.80E-06 | 3.70E-06 |
| hsa04390 | Hippo signaling pathway | 121/4708 | 157/8105 | 3.72E-07 | 6.80E-06 | 3.70E-06 |
| hsa04310 | Wnt signaling pathway | 127/4708 | 166/8105 | 3.99E-07 | 6.91E-06 | 3.76E-06 |

**Supplementary Table -4a:** Significant pathways between TA and Healthy controls in γδ T-cells identified by GO database

| **ONTOLOGY** | **ID** | **Description** | **setSize** | **enrichmentScore** | **NES** | **pvalue** | **p.adjust** | **qvalues** |
| --- | --- | --- | --- | --- | --- | --- | --- | --- |
| BP | GO:0050852 | T cell receptor signaling pathway | 106 | 0.555852 | 2.28751 | 7.21E-10 | 2.56E-06 | 2.49E-06 |
| BP | GO:0036230 | granulocyte activation | 283 | -0.49425 | -1.80552 | 8.24E-10 | 2.56E-06 | 2.49E-06 |
| BP | GO:0042119 | neutrophil activation | 281 | -0.49706 | -1.81456 | 1.27E-09 | 2.63E-06 | 2.56E-06 |
| BP | GO:0002283 | neutrophil activation involved in immune response | 275 | -0.49108 | -1.79304 | 2.67E-09 | 4.15E-06 | 4.04E-06 |
| CC | GO:0030141 | secretory granule | 449 | -0.44707 | -1.66153 | 4.57E-09 | 4.58E-06 | 4.46E-06 |
| BP | GO:0043312 | neutrophil degranulation | 273 | -0.49317 | -1.79789 | 4.79E-09 | 4.58E-06 | 4.46E-06 |
| BP | GO:0045055 | regulated exocytosis | 440 | -0.44698 | -1.66078 | 5.16E-09 | 4.58E-06 | 4.46E-06 |
| BP | GO:0002274 | myeloid leukocyte activation | 366 | -0.46345 | -1.71296 | 7.52E-09 | 5.85E-06 | 5.69E-06 |
| BP | GO:0002275 | myeloid cell activation involved in immune response | 304 | -0.48046 | -1.75865 | 9.21E-09 | 6.36E-06 | 6.19E-06 |
| BP | GO:0002446 | neutrophil mediated immunity | 282 | -0.48632 | -1.77585 | 1.19E-08 | 7.29E-06 | 7.09E-06 |
| BP | GO:0043299 | leukocyte degranulation | 300 | -0.47919 | -1.75615 | 1.29E-08 | 7.29E-06 | 7.09E-06 |
| BP | GO:0030217 | T cell differentiation | 155 | 0.467159 | 2.08781 | 3.00E-08 | 1.54E-05 | 1.50E-05 |
| BP | GO:0002444 | myeloid leukocyte mediated immunity | 311 | -0.46783 | -1.71453 | 3.23E-08 | 1.54E-05 | 1.50E-05 |
| BP | GO:0042110 | T cell activation | 274 | 0.376926 | 1.770238 | 4.46E-08 | 1.98E-05 | 1.93E-05 |
| CC | GO:0042101 | T cell receptor complex | 21 | 0.83443 | 2.508667 | 6.42E-08 | 2.66E-05 | 2.59E-05 |
| BP | GO:0002250 | adaptive immune response | 225 | 0.405206 | 1.882596 | 7.25E-08 | 2.81E-05 | 2.74E-05 |
| BP | GO:0030098 | lymphocyte differentiation | 218 | 0.406068 | 1.898771 | 7.69E-08 | 2.81E-05 | 2.74E-05 |
| BP | GO:0050851 | antigen receptor-mediated signaling pathway | 136 | 0.472666 | 2.063648 | 1.02E-07 | 3.51E-05 | 3.42E-05 |
| BP | GO:0006887 | exocytosis | 498 | -0.4224 | -1.57201 | 2.37E-07 | 7.74E-05 | 7.54E-05 |
| BP | GO:0002366 | leukocyte activation involved in immune response | 392 | -0.43644 | -1.61647 | 2.76E-07 | 8.59E-05 | 8.36E-05 |

**Supplementary Table -4b:** Significant pathways between TA and Healthy controls in γδ T-cells identified by KEGG database

| **ID** | **Description** | **GeneRatio** | **BgRatio** | **pvalue** | **p.adjust** | **qvalue** |
| --- | --- | --- | --- | --- | --- | --- |
| hsa04510 | Focal adhesion | 143/3623 | 201/8105 | 1.48E-14 | 4.88E-12 | 2.60E-12 |
| hsa04152 | AMPK signaling pathway | 91/3623 | 120/8105 | 2.74E-12 | 4.51E-10 | 2.41E-10 |
| hsa04919 | Thyroid hormone signaling pathway | 90/3623 | 121/8105 | 2.34E-11 | 2.57E-09 | 1.37E-09 |
| hsa01521 | EGFR tyrosine kinase inhibitor resistance | 62/3623 | 79/8105 | 7.51E-10 | 6.18E-08 | 3.30E-08 |
| hsa04015 | Rap1 signaling pathway | 137/3623 | 210/8105 | 1.04E-09 | 6.82E-08 | 3.64E-08 |
| hsa04151 | PI3K-Akt signaling pathway | 212/3623 | 354/8105 | 3.21E-09 | 1.52E-07 | 8.14E-08 |
| hsa04660 | T cell receptor signaling pathway | 76/3623 | 104/8105 | 3.36E-09 | 1.52E-07 | 8.14E-08 |
| hsa05135 | Yersinia infection | 95/3623 | 137/8105 | 3.74E-09 | 1.52E-07 | 8.14E-08 |
| hsa04928 | Parathyroid hormone synthesis, secretion and action | 77/3623 | 106/8105 | 4.17E-09 | 1.52E-07 | 8.14E-08 |
| hsa04010 | MAPK signaling pathway | 179/3623 | 294/8105 | 1.00E-08 | 3.31E-07 | 1.77E-07 |
| hsa04071 | Sphingolipid signaling pathway | 83/3623 | 119/8105 | 2.38E-08 | 7.11E-07 | 3.80E-07 |
| hsa04012 | ErbB signaling pathway | 63/3623 | 85/8105 | 3.03E-08 | 8.30E-07 | 4.44E-07 |
| hsa04810 | Regulation of actin cytoskeleton | 137/3623 | 218/8105 | 3.66E-08 | 9.26E-07 | 4.95E-07 |
| hsa04211 | Longevity regulating pathway | 65/3623 | 89/8105 | 4.81E-08 | 1.13E-06 | 6.04E-07 |
| hsa04611 | Platelet activation | 85/3623 | 124/8105 | 5.73E-08 | 1.26E-06 | 6.72E-07 |
| hsa04722 | Neurotrophin signaling pathway | 81/3623 | 119/8105 | 1.89E-07 | 3.80E-06 | 2.03E-06 |
| hsa04140 | Autophagy - animal | 91/3623 | 137/8105 | 1.97E-07 | 3.80E-06 | 2.03E-06 |
| hsa04014 | Ras signaling pathway | 142/3623 | 232/8105 | 2.20E-07 | 4.01E-06 | 2.14E-06 |
| hsa04068 | FoxO signaling pathway | 87/3623 | 131/8105 | 3.68E-07 | 6.37E-06 | 3.41E-06 |
| hsa04931 | Insulin resistance | 74/3623 | 108/8105 | 4.25E-07 | 6.99E-06 | 3.73E-06 |
