# Supplementary material for: Genome-Wide DNA Methylation Profiling in CD8 T-Cells and Gamma Delta T-Cells of Asian Indian Patients With Takayasu Arteritis
Source: Front Cell Dev Biol. 2022 Jun 23;10:843413. doi: 10.3389/fcell.2022.843413 (PMC9259853; doi:10.3389/fcell.2022.843413)
Supplement: Supplementary file 2 [file DataSheet1.pdf]

Supplementary Figures

Supplementary Figure S1. Representative picture showing density plot before and after normalisation in the type-II probe.

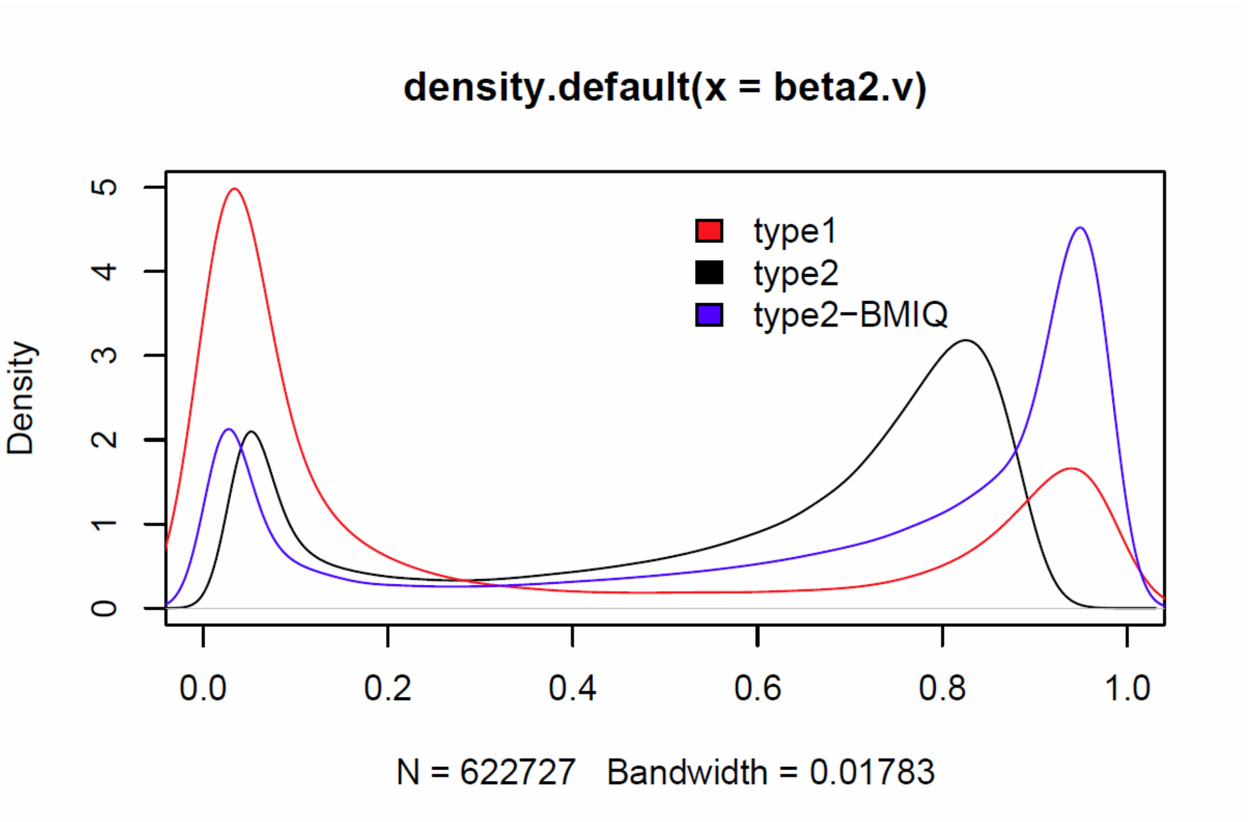

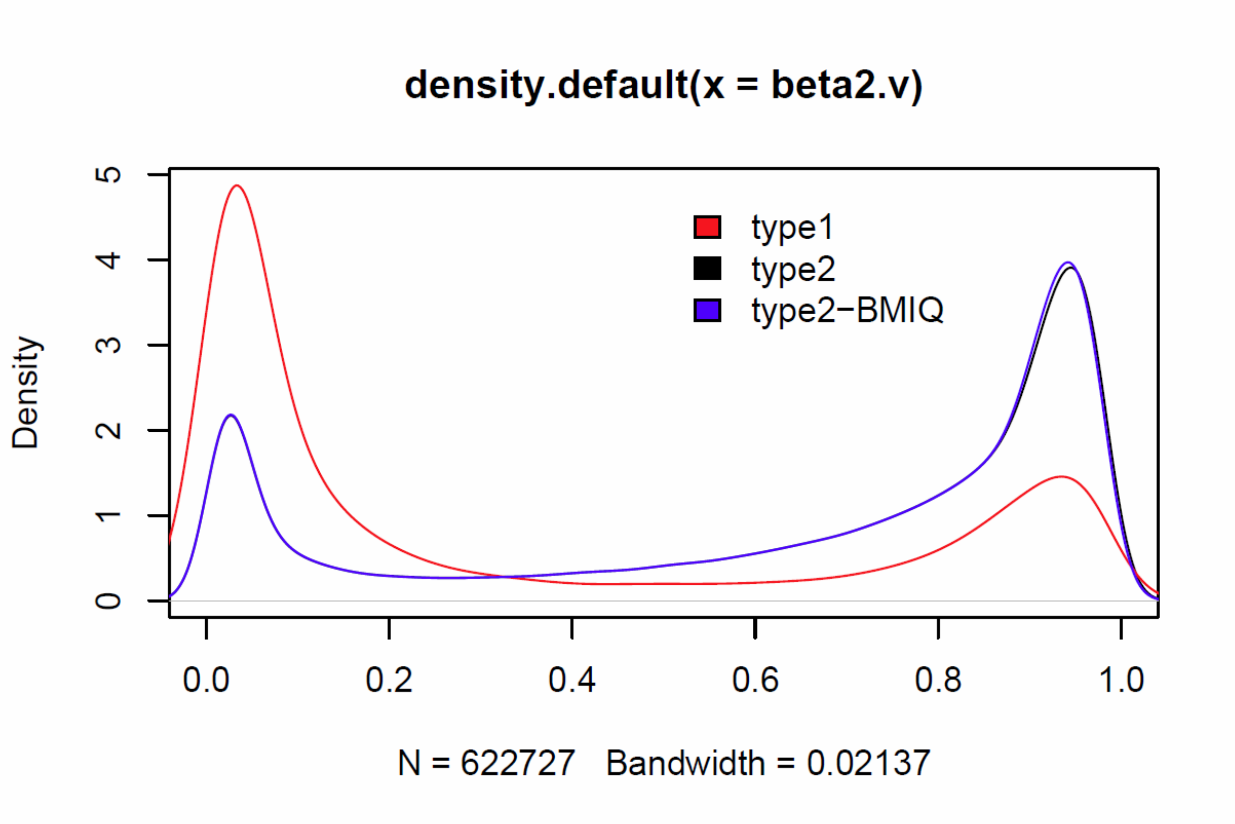

Supplementary Figure S2. a. GO and b. KEGG term enrichment analyses for the differentially methylated regions in CD8 T cells of patients with TA on comparing healthy controls. Plot showing activated and suppressed were interpreted as hypermethylated and hypomethylated respectively.

Supplementary Figure S2a.

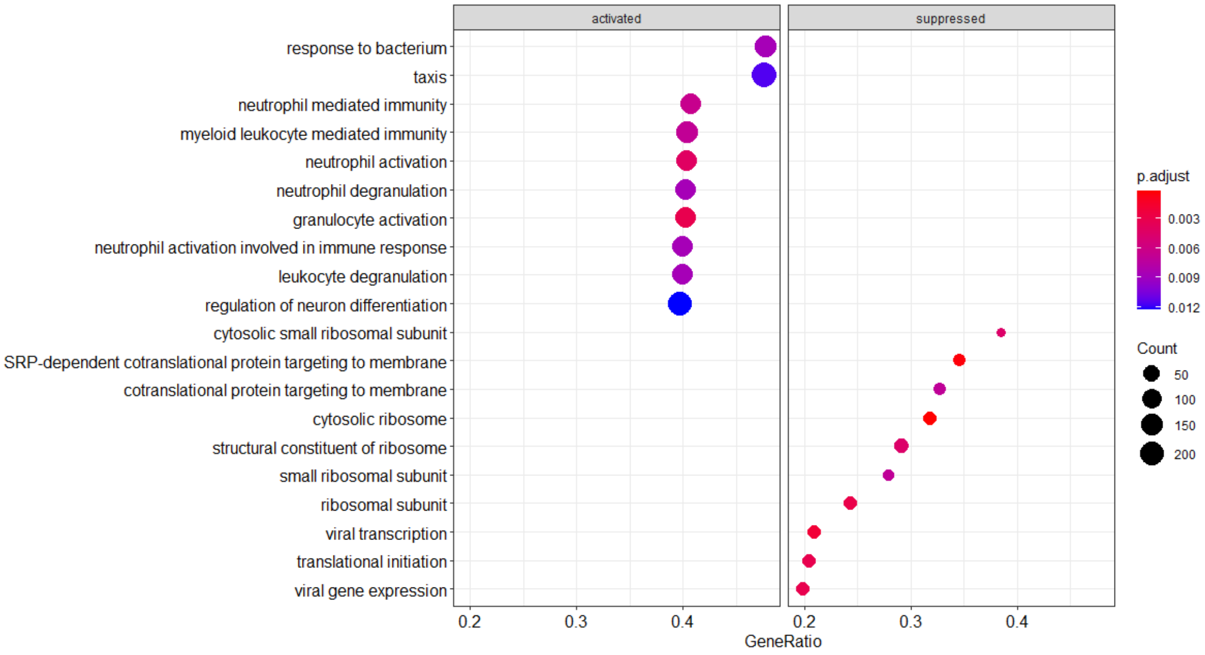

Supplementary Figure S2b.

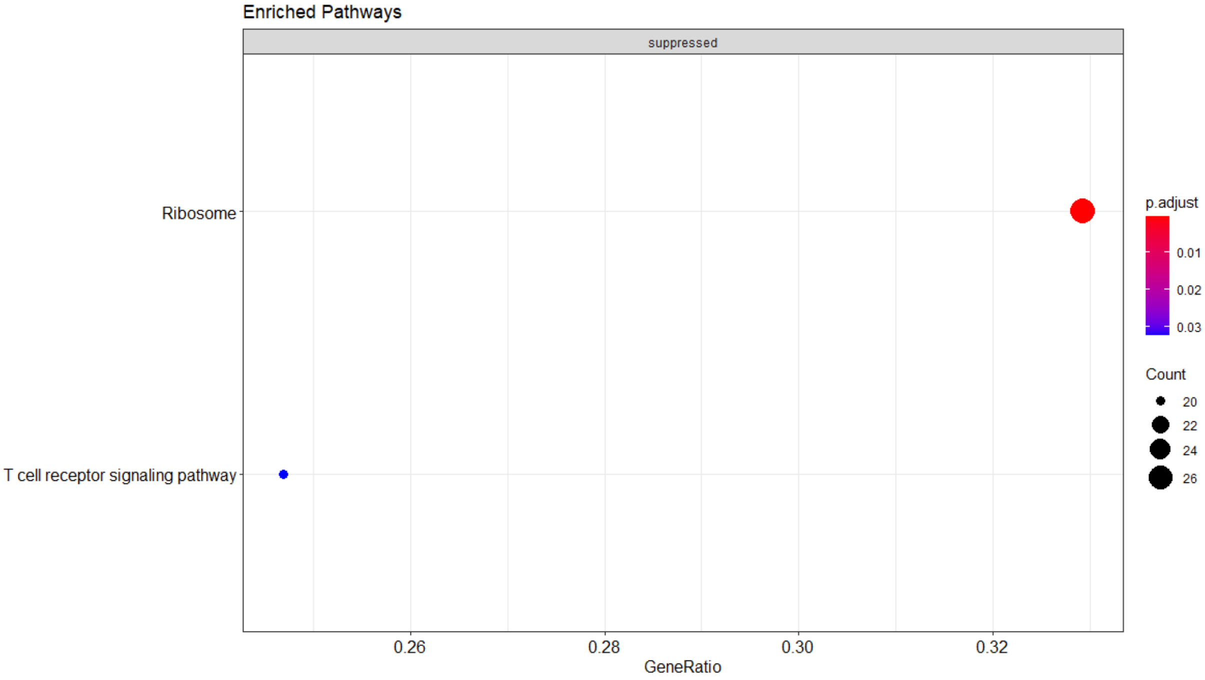

Supplementary Figure S3.a. GO and b. KEGG term enrichment analyses for the differentially methylated regions in γδ T cells of patients with TA on comparing

healthy controls. Plot showing activated and suppressed were interpreted as hypermethylated and hypomethylated respectively.

Supplementary Figure S3a.

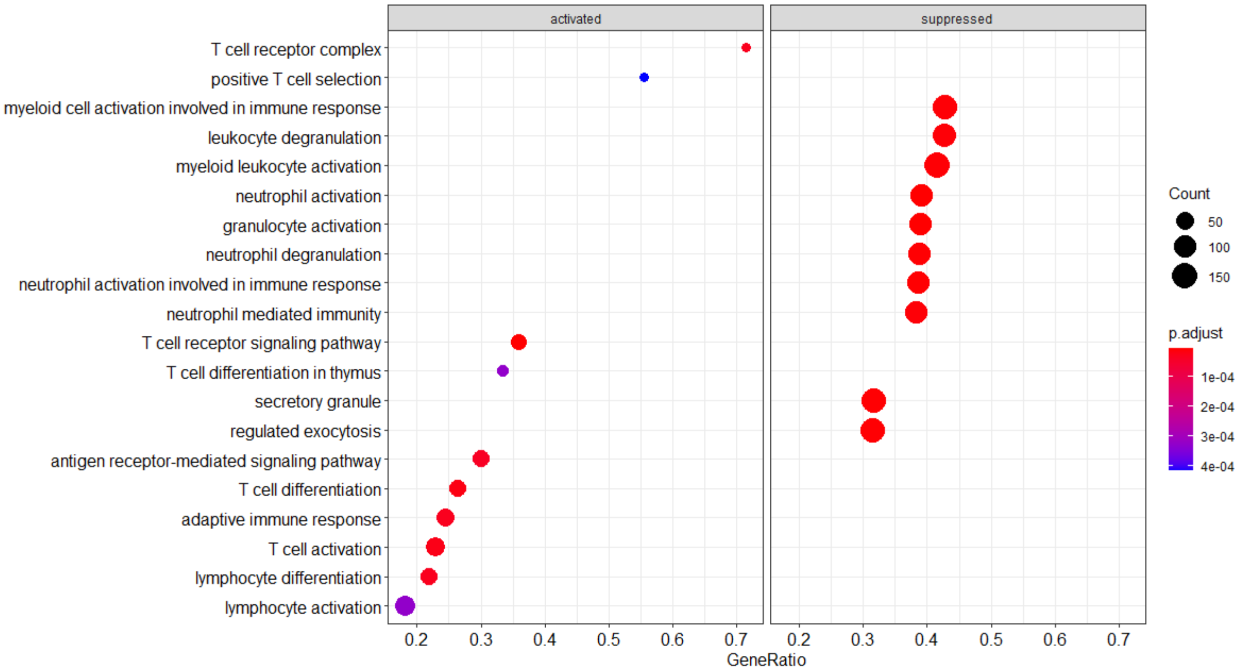

Supplementary Figure S3b.

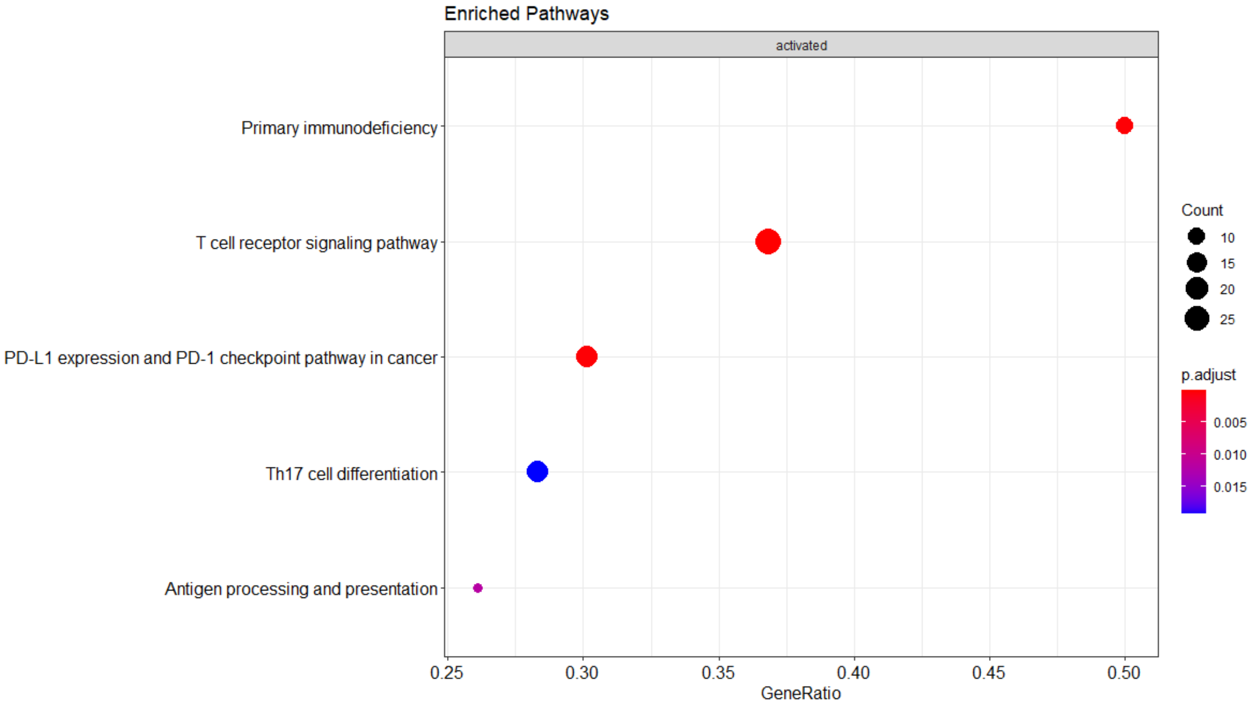

Supplementary Figure 4 .a. TCR signalling pathways showing hypomethylated and hypermethylated genes in CD8 T cells.

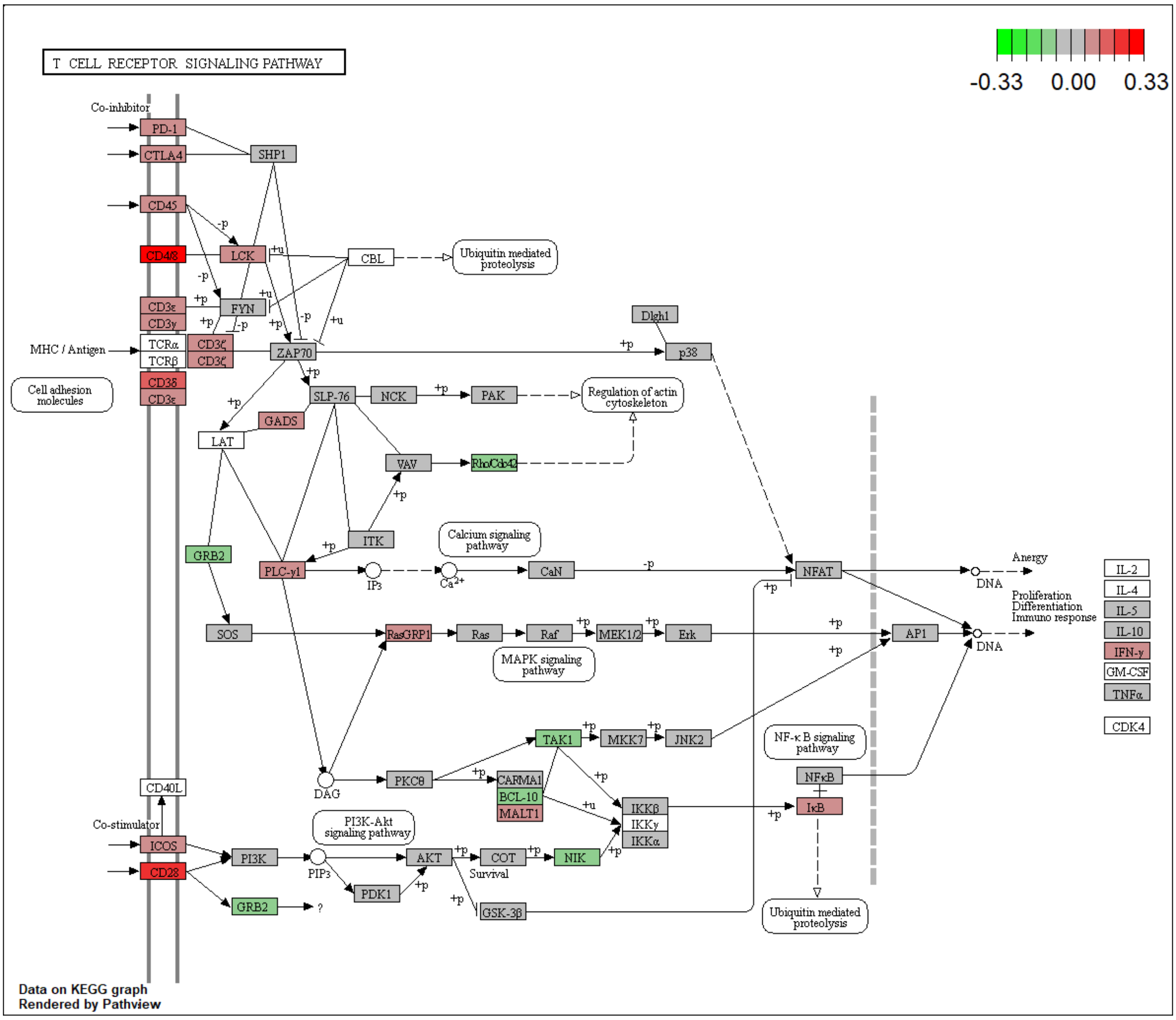
T CELL RECEPTOR SIGNALING PATHWAY
-0.33 0.00 0.33
Co-inhibitor
PD-1
CTLA4
CD45
CD4/8
CD3ε
CD3γ
MHC / Antigen
TCRα
TCRβ
CD3ζ
CD3δ
Cell adhesion molecules
SHP1
LCK
FYN
CBL
Ubiquitin mediated proteolysis
ZAP70
Dlgh1
p38
SLP-76
NCK
PAK
Regulation of actin cytoskeleton
GADS
LAT
VAV
Rho/Cdc42
ITK
Calcium signaling pathway
GRB2
PLC-γ1
IP3
Ca2+
CaN
NFAT
Anergy
DNA
Proliferation
Differentiation
Immuno response
IL-2
IL-4
IL-5
IL-10
IFN-γ
GM-CSF
TNFα
CDK4
SOS
RasGRP1
Ras
Raf
MEK1/2
Erk
AP1
MAPK signaling pathway
TAK1
MKK7
JNK2
NF-κ B signaling pathway
NFκB
IκB
DAG
PKCθ
CARMA1
BCL-10
MALT1
IKKβ
IKKγ
IKKα
CD40L
Co-stimulator
ICOS
CD28
PI3K
PIP3
PI3K-Akt signaling pathway
AKT
COT
NIK
Survival
PDK1
GSK-3β
?
Data on KEGG graph
Rendered by Pathview

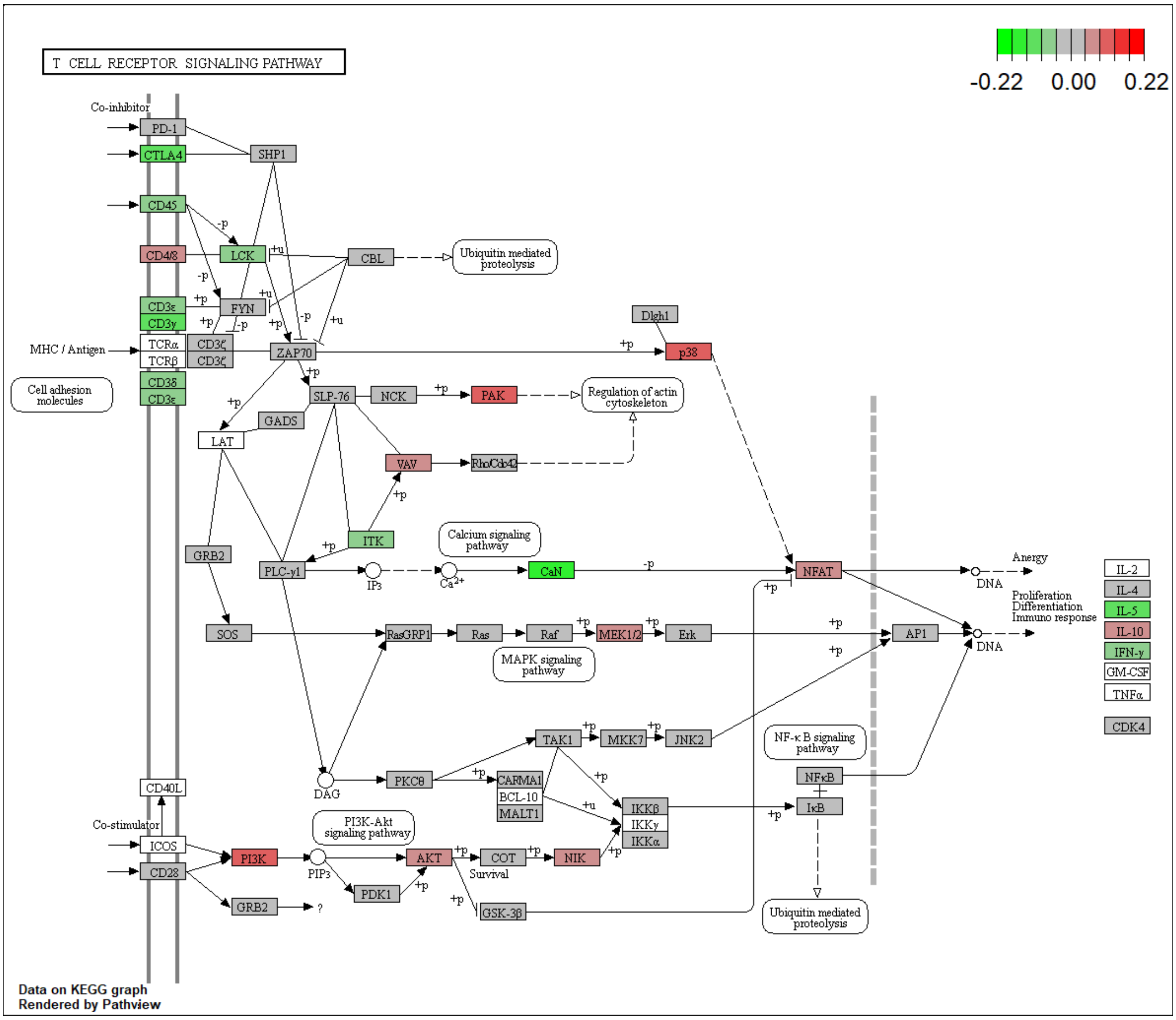

Supplementary Figure 4. b. TCR signalling pathways showing hypomethylated and hypermethylated genes in γδ T cells.
